# Potential fish yield and biology of some fish in Lake Hertale, Afar regional state, Ethiopia

**DOI:** 10.1016/j.heliyon.2023.e18661

**Published:** 2023-07-29

**Authors:** Alemayehu Wubie, Kibru Teshome, Gashaw Tesfaye

**Affiliations:** Ethiopian Institute of Agricultural Research (EIAR), National Fishery and Other Aquatic Life Research Center, P.O. Box-64, Sebeta, Ethiopia

**Keywords:** Lake Hertale, Macrophytes, Tilapia, Catfish and *Lebobarbus* and common carp

## Abstract

The Afar region is characterized by an arid and semi-arid climate with erratic rainfall and is prone to frequent droughts despite being endowed with fresh and saline water bodies along the Awash River Basin. Lake Hertale is one of those fresh water bodies located in the middle of the Awash River Valley. The lake's fishery is presumed not to have been exploited before. The study attempted to describe some physico-chemical parameters, fish species composition, estimate production potential which are relevant to develop a lake fishery with the prospect of contributing to food and nutrition security. The nutrient and chlorphyl-*a* measurements indicated eutrophic state but with apparently comparative low primary biomass. Conductivity, dissolved oxygen and temperature were within the optimal range for warm water species along with slightly alkaline pH. It was found that Lake Hertale is home to four important fish species, namely, Nile Tilapia (*Oreochromis niloticus*)*,* barbs (*Lebobarbus intermidus*), African catfish (*Clarias gariepinus*) and Common carp (*Cyprinus carpio*), Nile crocodiles, several species of birds and hippopotamus. *Labeobarbus* constituted 56%, followed by Nile tilapia with 31% of the total population. The African Catfish constituted only 9% of the catch. The mean lengths of African Catfish and *Labeobarbus were* 47.2 and 38 cm respectively. The mean lengths of Nile tilapia and Common carp were 22.8 and 21.7 cm respectively. The fish production potential of the lake was estimated from 94.3 to 238 tons per year, with an average production of 145.7 tons per year. The estimate production potential can support up to 21 to 28 fishers, with an average number of 25 fishermen to sustainably utilize the lake's fishery potential. Lake Hertale and other adjacent lakes in the Afar region of Ethiopia can complement to the fight against hunger and malnutrition. Besides, through appropriate fishery development intervention, it is possible to create employment for the predominantly pastoralist Afar community.

## 1 Introduction

Lake and reservoir fisheries play important role in livelihood improvements for the rural community in the form of fish for household consumption and income generation, particularly in places where other alternative sources of income are limited [1,2]. According to ref. [3] report, capture-based fishing employs around 39.1 million people, some on a full-time basis, some on a part-time basis, and others on an occasional basis. Most of these activities take place in underdeveloped nations the vast majority of which are artisanal fisheries operated on a small scale. Developing countries raised their per capita fish consumption from 5.2 kg in 1961 to 19.4 kg per capita in 2017 [3].

Fish consumption in the least developed countries (LDCs) has increased by 1.3% per year. However, the [4] report indicates that Ethiopia has the lowest per capita fish consumption in sub-Saharan African countries, with 240 g per person per year. In Ethiopia, inland water fisheries such as lakes, reservoirs, wetlands, and swamps account for the majority of the capture-based fishery. Ethiopia's current captured-based fishery production potential is estimated at around 94,541 tons per year [5]. The future fish demand in the country is projected to reach 117,586 tons in 2025 [4]. Despite this, earlier reports indicate the country's fishery production is 35% utilized [4,6]. Later [7], estimated a 20% utilization from the potential whilst more recently, data from World Bank and FAO suggested annual production of 60002 tons/year, a 63% utilization of the estimated production potential [8].

Nevertheless, commercial capture fishery production is concentrated in accessible water bodies and market options, primarily in the Rift Valley lakes Koka, Ziway, Langano, Hawassa, Chamo, and Abaya, as well as the Northern Lakes Tana, Hyiq, and Ardibo. Lakes and reservoirs located in remote and inaccessible areas support sizable subsistence fisheries yet production levels largely unaccounted. In particular, lakes and reservoirs in the Afar region are least known and underutilized except for Tendaho Reservoir [9]. A recent report on water resources survey in the Afar region, a home to around 2 million people [10], indicates that there are potential lakes and reservoirs of fisheries potential all of which are located in the middle and lower Awash River basins [9,11]. These include Lake Hertale, Yardi, Liado, Gammari, and Afambo. Tendaho and Kesem reservoirs, constructed primarily for irrigation of sugarcane plantations, complement to the fishery potential in Afar region. Based on empirical models [7], estimated the total fish production potential of water bodies in the Afar Region to be 2656 tons per year excluding the recently built Kesem irrigation reservoir. The water resources in the region can provide further employment and income to the riparian pastoralist communities through development of sustainable fisheries. The Awash River is the main water supply to each of the lakes except Lake Hertale and Kesem reservoir. Lake Hertale itself is an exorheic lake which has possible subterranean connection with Awash River based on authirs’ field observation.

A previous socio-economic survey indicated that there was a high fish demand in the Afar region mainly from truck drivers stopping in towns along a vital transport route to Djibouti, despite local pastoralists prefer canned fish [12]. A recent study by Ref. [13] indicated an increase in fish consumption and positive perception to fish as food after fish production begun in Tendaho reservoir. The growing fish demand can be met by integrating capture-based fishery with irrigation farming activities in the region. Nevertheless, there are no serious consideration of the fishery sector as source of income and employment as of now due to possibly inaccessibility of roads and remoteness of many of the water bodies and predominantly pastoralist lifestyle with subsequent difficulties in reaching out the communities with extension services. On the other hand, there is no scientific information on physico-chemical features and fish biology of L. Hertale in particular apart from a recent general survey for waterbodies of fisheries importance [11]. Thus, the present study aims to provide first description of some biological aspects of fish species, fish production potential and general physico-chemical features of L. Hertale and highlight the need for promoting fishery as a source of food, income and employment.

## 2 Materials and methods

### 2.1 Description of study site

**Figure 1:**
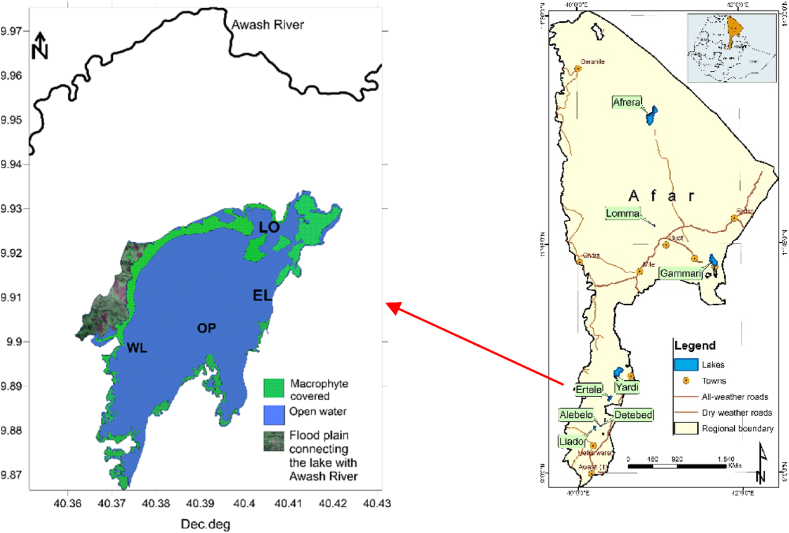
Map indicating Lake Hertale in Southern Afar Regional State, Ethiopia

Lake Hertale is located in the west part of Meteka sub-urban in Gewane district, Afar Region (09° 54.577′ N; 040° 24.278′ E). It is 18 km West of Meteka Town at an altitude of 579 m.a.s.l. Lake Hertale and another adjacent Lake Yardi are located in middle part of Awash River basin, while the Lower Awash Basin includes Lakes Gammari, Afambo and Abe. The total surface area is 14 km^2^. The South-Eastern part of the lake is surrounded by steep rocky heels. The lake's North-East and West shores are flat and extensively covered with macrophytes (Fig. 1). The Lake is believed to have subterranean or subsurface connection to Awash River along the North-East which is also extensively covered by macrophytes (Fig. 1). Apart from this, there are no apparent surface inlet or outlet rivers/streams connecting the lake with Awash River. The lake is also fed by numerous hot springs around the shore complementing runoff. The adjacent River Awash originates from central west part of Ethiopia and has 1200 km length and terminates in Lake Abe. The river is home to wildlife such as Nile Crocodiles, hippopotamus, and other mammals and several aquatic bird species which was reason for establishment of the adjacent Awash National Park. The study included 4 sampling sites, namely, Eastern littoral (EL), Western littoral (WL), pelagic (OP) and lake outlet (LO; i.e, region of the lake presumed subterranean to have connection to Awash River; Fig. 1).

### 2.2 Physico-chemical parameters

*In situ* water physico-chemical variables were recorded at four representative sites, namely Eastern littoral, Western littoral, Pelagic and Lake outlet. Dissolved oxygen (DO), pH, conductivity, water temperature and salinity were measured in vertical depth intervals using a digital multi-meter probe (Model HQ40d, HACH). Water transparency was measured with Secchi disk of 20 cm in diameter. Lake depth was measured at designated points using digital eco-sounder. Water samples were collected for the analysis of major dissolved inorganic nutrients (phosphate, nitrate and total ammonium). Water samples were filtered with a 0.45 μm GF/C (Whatman) for determination of dissolved inorganic nutrients. Soluble reactive phosphorus (SRP) and total phosphorous (TP) was determined Spectrophotometrically using ascorbic acid method (the latter after digestion with potassium-perusulphate), total ammonium_nitrogen (TAN), nitrite-nitrogen and nitrate-nitrogen were determined by indophenol blue method [14]. Water samples were taken at a depth of 0.5 m for chlorophyll *a* determination. A known volume of water sample was filtered through GF/C (Ederol) filter paper. The filtered paper material was kept in a vial coiled with aluminum foil in an ice box. Wet extraction of chlorophyl *a* was conducted by dissolving a ground filter material in to a final volume of 10 ml of 90% acetone. From the clear solution, the absorbance of the pigment was measured spectrophotometrically at 665 mm. The concentration of chl-*a* was estimated using the equation from [15].

$$Chl-a\;\left(\mu gl^{-1}\right)=\frac{11.4*665abs*extract\left(ml\right)}{Filtrate\left(l\right)*d}$$

where filtrate is the filtered volume of water and is cuvettes path length in cm, 665 abs stands for absorbance value at 655 nm wavelength after blanking with 90% acetone.

### 2.3 Fish production potential

The lake's fish production potential was estimated using three different empirical models.

Model I This was based on Morpho-Edaphic Index (MEI) [16].

Log_10_Y = 0.942 + 0.3813*Log_10_ MEI; r = 0.51 [16].

where; Y = the yield in kgha^−1^year^−1^ and MEI = the Morpho-Edaphic Index expressed in μS/cm which is calculated as follows

$$MEI=\frac{Conductivity\;in\;\mu S/cm}{Mean\;depth\;in\;M}$$

Model II : A further improvement was done in fish yield model using selected African Lakes and reservoirs [17]. improved the relationship by including lake area and fishery yield is calculated as follow

[17] Log_10_Y = 1.4071 + 0.3697.Log10.MEI-0.00005465A; R = 0.81----------------------------

Model III: This model was derived by Ref. [18] based on relationship between catch and area of 46 lakes and 25 reservoirs in Africa: Y = 8.32. A^0.92^R = 0.964---------------------------------------------------- [18].where Y = the total yield in tons per year and A = lake area in square kilometers.

Fish sampling: The Eastern littoral (EL) and open water (OP) sites were selected for fish sampling to represent the littoral and open water region of the lake (Fig. 1). Fish samples were collected using gillnets (6 cm, 8 cm, 10 cm and 12 cm stretched mesh size) which were set overnight and caught fishes were collected from each net and kept in separate polyethylene bags. The total lengths (TL), fork lengths for Common carp and barbs and total body weight (TW) of each specimen were measured. Sex identification was done after dissection and maturity level of some fish specimen were rated based on five point's maturity level determination [19] and each gonad was removed and weighed to the nearest 0.1 g.

### 2.4 Length at first maturity

The length at first maturity (L_50_), length at which 50% of the total number of individuals of a length group reaches maturity [20], was estimated using a logistic regression model.

### 2.5 Name of the ethical committee

As per the request, we are listed down the full name of the ethical research committee that approved this research.

1. Dr. Marshet Adugna-chair person
2. Habtamu Tadesse-member
3. Seferu Tadesse-member
4. Fekadu Tefera-member

## 4 Results and discussion

### 4.1 Morphometric observation

Lake Hertale is a shallow lake with an average depth of 3.7 m and maximum depth of 4.6 m. Based on visual observation, the lake is appears to be fed primarily with hydrothermal hot springs and ground water seepage and precipitation. The estimated total area covered by macrophytes is 3.8 km^2^, and about 1.34 km^2^ is a floodplain that connects the lake and the river Awash (Fig. 1). The local communities mostly used the lake for livestock watering and bathing. Fishing was nonexistent due to superstitious beliefs. Besides, the lake is the home to wild life including crocodiles, hippopotamus, different aquatic birds, and desert warthogs.

### 4.2 Physico-chemical parameters

**Fig. 2:**
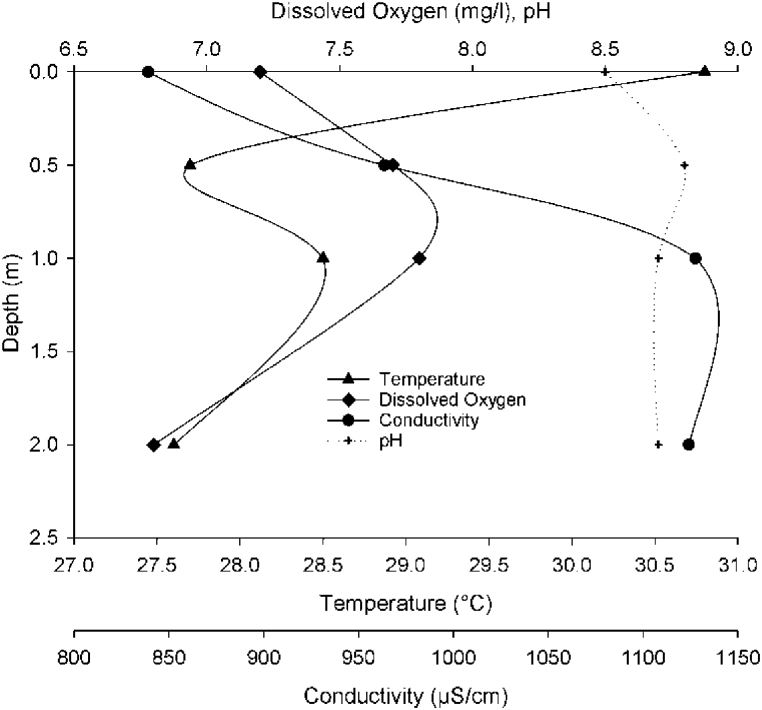
Vertical profiles of some physico-chemical parameters of Lake Hertale.

Lake Hertale is moderately turbid with a secchi depth transparency (SDT) of 0.7 m which can be attributed to the lack of apparent surface inflows that could bring sediment. Besides, the north-western shore is covered with wetland macrophytes dominated by Cyprus papyrus, Phragmites sp., Typha sp., and a variety of aquatic and floodplain plant species which possible act as filter zone [21]. The mean dissolved oxygen content in Lake Hertale was 7.4 mg/l and the maximum was 7.8 mg/l, which is optimal for growth and survival of most warm water fish species [22]. The pH values ranged between 8.5 and 8.8 with no variation along the water column (Fig. 2) which is generally suboptimal for aquatic life [23] and has no effect on Nile Tilapia performance [24]. The mean Conductivity was 1013.7 μS/cm which is slightly saline yet lies within the range of fresh water. Conductivity is highly associated with temperature, alkalinity, total hardness, calcium, total solids, total dissolved solids, chemical oxygen demand, and iron concentration in the water [21].

The surface water temperature values ranged between 27.6 and 30.8 °C with a mean value of 28.7 °C attributed to the semi-arid climate of the region and shallow depth. The temperature value represents an optimum range for growth and reproduction of warm water fish species such as *O. niloticus and C. gariepenis* [22]. Water temperature affects fish growth, reproduction and immunity in aquatic ecosystems [25]. The water temperature controls the rate of all chemical reactions and fish growth, reproduction and immunity in aquatic ecosystems since rate of biological and chemical processes depend on water temperature.

Nutrient and chlorophyl-*a* content indicate the lake is still eutrophic, in contrast to a hyper-eutrophication trend in the adjacent Ethiopian Rift Valley lakes [26]. There is no remarkable spatial difference in the values of total ammonium nitrogen (TAN), nitrite-nitrogen, nitrate-nitrogen, total phosphorous (TP) and chlophyl-*a* (Table 1). Chlorophyl-*a* is much lower than lakes in the Ethiopian Rift Valley such as L. Zeway, which measured up to 82 μg/l in the 1990's [27].

**Table 1:** Composition of some dissolved inorganic nutrients and primary biomass in Lake Hertale.

Sampling sites	NO_2_–N (mg/l)	NO_3_–N (mg/l)	NH_4_–N (mg/l)	TP (mg/l)	Chl*a* (mg/l)	Tot. Alkalinity (meq/L)
Eastern littoral	0.588	0.626	0.027	0.138	0.0176	8.6
Western littoral	0.589	0.628	0.031	0.207	0.016	5.2
Pelagic	0.589	0.619	0.035	0.265	0.0189	7.4
Lake outlet	0.591	0.561	0.027	0.140	0.0181	7.6

### 4.3 The fish production potential

Estimating the fish production yield is important in fisheries management to make a more accurate appraisal of the expected fish harvest from the lake and reservoirs [28]. The estimated mean annual fish production yield in Lake Hertale is 145.7 tons per year which is higher than a previous estimates of 59 tons/year by Ref. [29] and 79 tons/year by Ref. [5]. Other water bodies in the Afar region with fish production potential include, Lake Gammari (635.7 tons per year) Lake Yardi (715 tons per year) Tendaho (1414.7 tons per year) [5] implying that the Afar Region is endowed with huge fish resource potential which would contribute to food security, combating hunger and malnutrition (Table 2). According to Ref. [30] the mean annual catch of African Lakes and reservoirs per fishermen has been 3 ton/year as a maximum catch limit while [31] suggested the optimum yields of African lakes and reservoirs are attained with a fishing density of 1–2 fishermen/km2. Lake Hertale's estimated yield of 145.7 tons/year can employ 25 to 48 fishermen without exceeding its maximum sustainable exploitation level.

**Table 2:** Mean annual potential fish yields as estimated using empirical models in some lakes in Afar Regional State.

Lakes	Model I PotentialYield (t/y)	Model II Potential Yield (t/y)	Model III Potential Yield (t/y)	Average Potential Yield (t/y)
Lake Hertale	104.1	238	94.3	145.7
Lake Gammari	703	790	414	635.7
Lake Yardi	771.9	861.5	511.7	715
Tendaho	1547	1759	938	1414.7

The Afar Region is drought prone area and acute child malnutrition is higher than the national average [32]. On the contrary, that only about 21% of the actual potential of Tendaho Reservoir is currently being exploited by local pastoralists that have settled in the vicinity [13]. The large majority of pastoralists adjacent to the reservoir normally move with their cattle especially during the dry season. Evidences suggest that while farming might not be a promising venture in dry land and semi-arid environments, lake and reservoir fisheries are not only economically important but also leads to societal change through livelihood diversification and change in life style [33]. Therefore, development intervention is needed to develop fishery resources to utilize the potential resources and combat food hunger in the region.

### 4.4 Fish species composition

**Fig. 3:**
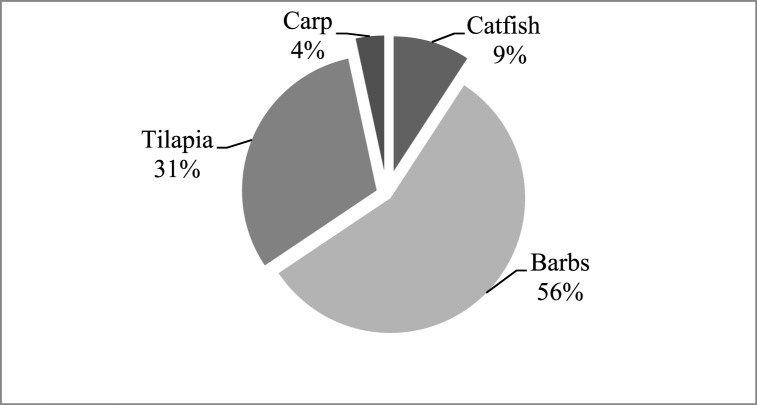
Fish catch percentage composition in Lake Hertale.

According to Ref. [34] the Rift Valley Lakes and Rivers are home to about 30 different fish species of which 11 species were recorded in the Awash River. An additional species was added to the last more recently [35] while further taxonomy studies are expected to raise the number of species. Among them are two species (*Garra makiensis* and *Voricohinus beso*) are believed to be endemic to the Awash Basin. In this study, a total of 1646 individuals of four fish species were caught, namely Nile Tilapia (Orechromis niloticus L), Common carp (*Cyprinus carpio*), catfish (*Clarias gariepinus* B) and barbus (Lebeobarbus intermedius) species. The first three species are commercially important. The catch was composed of, barbs (56%) of the total, tilapia (31%), catfish (9%) and Common carp (4%) (Fig. 3). The barbs and catfish populations in the lake were larger in mean length and body weight. However, barbs have the lowest market value but still can contribute to nutritional improvement in communities with few alternative protein sources.

The lake is presumably never been exploited before, yet the African catfish, measuring up to 101 cm maximum length, is suitable for human consumption and market. The mean lengths of Common carp and Nile tilapia were 21.7 ± 5.9 and 22.8 ± 4.8 respectively (Table 3) which is possibly related to relatively lower productivity of the L. Hertale with chlorophyl-*a* content of just 13.1–18.9 μg/l compared to adjacent lakes in the Ethiopian Rift Valley (e.g. L. Zeway; [27]). Such low algal productivity implies less food for planktivorous fish species such as *O. niloticus* thus limiting their growth [36]. African catfish species are facultative piscivores, feeding occasionally on small fish, zooplankton, and benthic organisms [37].

**Table 3:** Fish species, mean length and condition factor each fish in Lake Hertale.

Species	Common name	Mean length (cm)		
		n		Kn
*Oreochromis niloticus*	Tilapia	512	22.8 ± 4.8	1.33 ± 0.56
*Cyprinus carpio*	Common carp	54	21.7 ± 5.9	1.23 ± 0.17
*Clarias gariepinus*	Catfish	152	47.2 ± 13.9	0.81 ± 0.17
*Labeobarbus intermedius*	Barbs	928	38.0 ± 5.6	1.14 ± 0.5

Notes: n = samples, Kn = condition factor of each fish.

Sex ratio:During the study, a total of 1647 fish specimens were collected of which 147 were females and 799 were males. The remaining was not sexed and was not included in the sex ratio analysis. Males outnumbered females significantly (χ2, P < 0.01) in every species except for African catfish (Table 4). This might be due to the active movement of males in searching for mates and courtship that makes them vulnerable to fishing gill nets. A previous study in Lake Tana indicated the dominance of the male over female tilapia, which was attributed to the reproductive behaviors of the male fish [38]. Although, the sex ratio can vary among species, seasons, and years, generally, it is close to 1:1 [39].

**Table 4:** The sex ratio and the number of females and males caught in Lake Hertale.

Fish species	sex		Female: Male ratio	*χ*^*2*^	Sign.
	Female	Male			
*Clarias gariepinus*	94	50	1:0.5	1.0	0.000**
*Labeobarbus intermedius*	410	450	1:1.1	24.6	
*Oreochromis niloticus*	139	290	1:2.1	3.0	
*Cyprinus carpio*	4	9	1:2.3	26.5	
Total	647	799	1:2.2	55.1	

Note: ** highly significant (P < 0.001).

### 4.5 Length at the first maturity

**Fig. 4:**
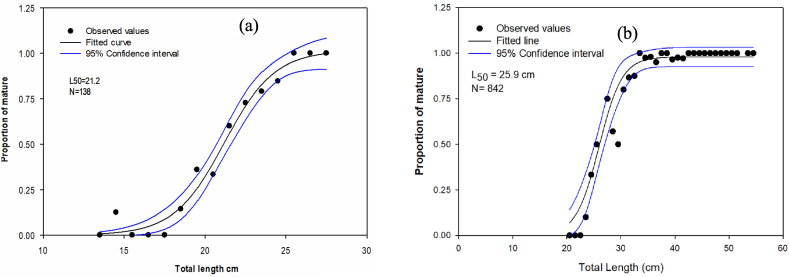
Size at first maturity for *O. niloticus* (a) and *L. intermedius* (b) in Lake Hertale. The blue lines are the 95% confidence interval of L50 and solid line (sigmoid curves) are fitted lines, black circles are observed proportion of matured fish. (For interpretation of the references to colour in this figure legend, the reader is referred to the Web version of this article.)

The length at which 50% of L. barbus fish first matured in Lake Hertale was 25.6 cm TL. Labeo.barbus is one of the commercially important fish species in the country and contributes up to 9% of the annual catch [5]. However, it was the least studied in Ethiopia. Our L_50_ value was lower than that of previous studies on Lake Koka (28.1 cm) [40]. But it was comparable to a study done in Lake Tana (25.9) [41] while the 95% confidence interval for the first maturity of *O. niloticus* was 21.2 cm in total length (Fig. 4). The smallest full-ripe female captured during the sampling period was 14 cm for O.niloticus and 23 cm for L.barbus. When fishing pressure increases *O*. niloticus tend to breed earlier and the size at first maturity decreases [42]. Except in Lake Chamo and Koka, the length at first maturity of O.niloticus in Lake Hertale is a bit greater than the rest of the Rift Valley Lakes of Ethiopia, attributed to presumed absence of exploitation (Table 2). The female O.niloticus fish length at first maturity in Lake Chamo was 42 cm [43], while in Lake Koka it was 24.6 cm [40] and in Alwero Reservoir, located in far West of the country, the female O.niloticus length at first maturity was 22.5 cm [44]. Tilapia is a warm-water fish that grows fast. Diet quality and digestibility have been one main determinant in the growth rate of tilapia and other fishes in general [45,46]. The growth rate in fish of same species in general is plastic because it differs in different water bodies [47]. Growth plasticity is particularly common in tilapia [48]. The growth rate can be different due to differences in water temperature, food quality and quantity, habitat size, and fishing pressure [49,50]. This indicates that environmental differences are more important than genetic ones in determining the growth rate of particular populations of tilapia that exist in different water bodies [51]. In the case of Common carp and catfish, length at first maturity was not estimated due to the small sample size.

## 5 Conclusion and recommendations

Although Lake Hertale is still in a eutrophic state, it is not as productive as several lakes in the Rift Valley in terms of primary biomass (chlorophyl-*a*) probably limiting the growth performance of Nile tilapia. Despite the comparably high nutrient content in L. Hertale, further observation is required to highlight factors limiting primary biomass compared to adjacent hyper-eutrophic lakes. Our study highlights the potential for fishery development around L. Hertale. Based on our limited sampling frequency and small sample size, the African catfish (*C. gariepinus*) is a better performing species in terms of body length. There are four species in L. Hertale three of which have commercial importance in the local market, namely Nile Tilapia, Common carp and African Catfish. A larger sample size would render a better estimation of length at first maturity [52].There are a number of fresh water bodies in the Afar Region with fishery potential that can provide the riparian community with high quality animal protein. The Afar people are predominantly pastoralists with no apparent cropping and fishing experience except around the Tendaho Resevoir. The local community's main staple foods are cereals and milk that don't suffice their protein requirements. There is also apparently a general lack of awareness on fishing, fish processing and the nutritional benefits of fish in the region. Hence, awareness should be created about the importance of fish to local households. In this regard, the fishing experience around Tendaho Reservoir should be extended to communities around Lake Hertale so that they benefit from the fishery sub subsector in terms of insuring food security and alternative sources of income. While the region is prone to drought and children are affected by malnutrition, we assert that utilizing the fishery resources will combat hunger and malnutrition in a community already vulnerable to impacts of climate change such as recurrent drought. Lake Hertale can contribute to food and nutrition security through appropriate development interventions and sustainable management of the lake's fishery afterwards.

## Ethics statement

The animal study was reviewed and approved by the animal ethics committee of the Fishery and Aquaculture Research Center (NFALRC).

## Author's contribution

AW: Conceptualization, field data collection, methodology, data processing, interpreting data and writing original draft; KT: Conceptualization, data collection and processing, reviewing and editing; GT: Data analysis, writing-review and editing. All authors agreed to be responsible for the content of the paper and approved it for publication.

## Data availability statement

Data will be made available on request.

## Funding

The research was funded by the 10.13039/501100004535Ethiopian Institute of Agricultural Research (EIAR), National Fishery and Aquatic Life Research Center (NFALRC), capture fishery research project.

## Declaration of competing interest

The authors declare that they have no known competing financial interests or personal relationships that could have appeared to influence the work reported in this paper.
